# Radon-222 Exposure and Dose Concentration Levels in Jordanian Dwellings

**DOI:** 10.1155/2020/6668488

**Published:** 2020-11-16

**Authors:** Akeel T. Al-Kazwini, Mohannad. M. Al-Arnaout, Tiba R. Abdulkareem

**Affiliations:** Department of Biomedical Engineering, School of Applied Medical Sciences, German Jordanian University, Amman 11180, Jordan

## Abstract

Exposure to high concentrations of radon gas is the leading cause of lung cancer for nonsmokers according to the World Health Organization (WHO) figures. With poor ventilation standards and lack of awareness among Jordanians, constant monitoring of radon concentrations is vital. Multiple efforts have been made since the 1990s in order to create a national radon map of Jordan, by acquiring average values of radon concentrations in major Jordanian cities. This study aims to replicate those efforts using a more accurate and modern way of detection for the purpose of comparing the current values with literature values and to update the previous radon concentration map of Jordan. The study concludes that radon concentrations in Jordan have mostly increased in the past 30 years from an overall average of 52 Bq/m^3^ to an average of 60.4 Bq/m^3^. Despite the increase, these results are considered under the threat line that is estimated conventionally by most of the international environmental and radiation-related organizations, which is 100–300 Bq/m^3^. It should be noted that only the Russeifa city has scored a value higher than the estimated threat line. This is due to the existence of abundant phosphate mines filled with condensed radon levels leaking from these ores. It is expected that radon concentrations in Jordan will increase in the coming years with the continuous urban sprawl and lack of public awareness about the radon gas health issue. A number of suggestions have been proposed in this study that could help the Jordanian society avoid a future possible health threat.

## 1. Introduction

Exposure to high levels of alpha energy due to radon (^222^Rn) gas concentration and its progeny is the main cause of lung cancer after smoking in the general population [1–3]. There are three main naturally occurring isotopes of radon: ^222^Rn, ^220^Rn, and ^219^Rn. Radon (^222^Rn) is a noble gas and part of the thorium-234 (^234^Th) and uranium-238 (^238^U) decay chain into stable lead-206 (^206^Pb). Radon's abundance depends on the amount of uranium that exists in the rock in the excavated sites [4]; both of uranium ores and uranium associated with phosphate ores are excavated in Jordan [5]. This means that the radon concentration levels will be higher in the excavated areas. The decay process takes millions of years, which means radon gas will always be abundant.

Previous studies proposed that the awareness rate in Jordan of radon exposure is very low and almost negligible [6]. Therefore, a thorough study and survey of the varying concentrations of radon gas in heavily populated dwellings in Jordanian provinces is needed. This study will shape a clear understanding of the local radon map, which will raise awareness among Jordanians about exposure and dose concentration levels.

Radon represents the greatest percentage of the natural indoor airborne radioactivity. Indoor air pollution has recently attracted a great deal of attention. With the trend towards reducing ventilation and infiltration rates in dwelling buildings, along with the popular use of granite in the indoor environment for its durability and decorative appearance, this problem has become even more serious [7–9].

Worldwide there is a variety of construction types where three main factors influence the choice of the construction type: economic aspect, environment, and well-being. Therefore, the construction types could be made mainly from concrete, clay brick, solid wood, wood frame, or steel frame structures [10, 11]. Knowing the construction type may help researchers to enhance their understanding of radon gas sources and its accumulation in the dwelling.

Buildings in Jordan are constructed mainly from concrete, covered from the outside with natural limestone blocks. The floor tiles of the buildings are made of various materials either ceramic, or marble, or mosaic (mosaic is made from aggregates of marble and granite mixed with concrete).

Local climate can also influence radon accumulation; in particular, humidity and temperature play a major role in building ventilation. Jordan has a mixed desert/Mediterranean climate, and hence, although it shows remarkable differences in temperature throughout the year, weather is not susceptible to extreme differences in humidity. [12].

Variation of radon concentration levels depends on many factors such as (1) construction type, (2) from season to season, (3) based on the floor level above the ground, (4) daily climate (i.e., pressure, humidity, and wind speed), (5) geographic and geological location, (6) area of uranium ores and uranium associated with phosphate ores, and (7) other factors [7, 8]. All of these factors can be found in Jordan in many dwelling areas throughout the kingdom [9].

The aim of this study is to measure the average values of ^222^Rn gas concentrations for various Jordanian provinces' dwellings. These values were statistically treated and compared with the literature values [6] for the purpose of updating the Jordanian radon map and to be a step ahead of any possible increase of radon exposure in Jordan. The study aims to determine whether the radon gas concentration values fall within the recommended acceptable range [4, 13–15] and to determine the dose risk level among Jordanians.

## 2. Materials and Methods

### 2.1. Selection of Measurement Locations

This study covers all Jordan provinces to shape a clear understanding of the local radon map, as well as the distribution of radon gas concentration in various multi-floor levels in dwellings for each province; 2–3 buildings located randomly were selected in each province. Radon measurements were carried out during fall and winter seasons of the year 2019. Indoor measurements were conducted at around 2 meter height and around 0.5 meter away from walls.

These cities were chosen for three main factors—heavy population, geographic and geological location, and rich phosphate deposits—*Amman, Irbid, Zarqa, Mafraq, Russeifa, Madaba, Karak, Ma'an, and Aqaba*. *Amman* is the capital city and is located in the central region of Jordan with elevation 600–1200 meter above the sea level. *Irbid* is located on the highland plateau in the north of the country. *Zarqa* is located in a desert area in the east of the country, and *Mafraq* is located in a desert area in the north east of the country. *Russeifa* consists of abundant phosphate mines and is located near Zarqa. *Madaba* is located in the west of the country near the Jordan valley fault, and *Karak* is located in the south west of the country also near the Jordan valley fault. *Ma'an* is located in a desert area in the south east of the country, and *Aqaba* is a coastal area located in the far south of the country.

In 2019, the reported percent relative humidity range in Jordan was between 51.0% and 24.0% and the reported annual average temperature in Jordanian dwellings was between 17°C and 25°C [12].

### 2.2. Mean Indoor Radon Concentrations in Dwellings

The indoor radon concentrations of six floor levels of dwelling buildings were measured. The buildings were built post-1980, constructed from concrete and covered from the outside with natural limestone blocks. The floor tiles of the buildings were made of various materials either ceramic, or marble, or mosaic (made from aggregates of marble and granite mixed with concrete).

The selected floor levels were the basement (B1), the ground floor (GF), the first floor (F1), the second floor (F2), the third floor (F3), and the fourth floor (F4). The basement level (B1) is the part of a building that is partly or completely below the level of the outside land. The ground floor level (GF) is the floor where the main entrance of the building is usually located and is at the level of the surrounding land; the levels F1 to F4 are sequential to these, giving six floors in total. The average household size was 4.8 people [16].

### 2.3. Dose Estimation

The annual effective dose (*E*) received by inhabitants was estimated in mSv/year from the values of radon concentration measured in the air using the following equation, as stated by the United Nations Scientific Committee on the Effects of Atomic Radiation (UNSCEAR 2000) [17] and based on dosimetry and epidemiological studies:

(1) $$\begin{array}{c} E=C*t*F_{\text{eq}}*9*10^{-6}, \end{array}$$

where

- *C*: Radon gas concentration in the dwelling air in Bq/m^3^.
(ii) *t*: Duration exposure time by inhabitants; in this study, a rounded occupancy factor of 0.8 is adopted, which is corresponding to 7000 hours (ICRP Publication 65, 1994) [14, 15].
(iii) *F*_eq_: Equilibrium factor for internal areas is the ratio of the equilibrium equivalent concentration to the radon gas concentration (i.e., a *F*_eq_ equals to 1 means full radioactive equilibrium between radon and its airborne short-lived progeny). The United Nations Scientific Committee on the Effects of Atomic Radiation (UNSCEAR) and the International Commission on Radiological Protection (ICRP) have adopted a typical worldwide *F*_eq_ factor of 0.4 for indoor air [13, 14, 17, 18]. Therefore, in this study, the *F*_eq_ factor of 0.4 was adopted. The conversion factor of 9 (nSv ∗ m^3^/Bq ∗ h) was used as recommended by the UNSCEAR (2000).

### 2.4. Radon Measurement Device and Method of Detection

In this study, a continuous portable digital radon gas detector (Corentium Home; Airthings), which is a particle (spectrometry) detector, was used to detect, track, and identify ionizing particles. It is based on a passive diffusion chamber with a silicon photodiode and alpha spectrometry. This type of detector is widely commercially used for radiation protection. The Corentium Home (Airthings) detector is likely to be the most competitive radon monitoring detector because of its features and cost; that is, this detector is easy to use and more selective to radon, has fast response time, operates on low battery power, and has low detection measurements with high accuracy. The device records every 1 hour and updates the daily average of short-term measurements (1–7 days), whereas for long-term measurements (months), the device updates and records the average every 1 day.

The measurement range of the detector is 0–9999 Bq/m^3^, and after 7 days of measurements at 100 Bq/m^3^, the standard deviations of accuracy and precision are lower than 10% [19, 20]. Warkentin et al. (2020) validated these values of the Corentium Home (Airthings) detector in a collaborative study between *Canadian Association of Radon Scientists and Technologists* and *Radiation Safety Institute of Canada* [21] in which an AlphaGuard DF2000 was used as the reference monitor with an error of ± 3% against a primary standard.

The test conditions inside the radon test chamber were within the temperature and humidity conditions inside a Jordanian dwelling during the testing period [12, 21]. The AlphaGuard DF2000 reference monitor was set at a radon concentration target of 200 Bq/m^3^, and the average radon concentration measured was 206 ± 28 Bq/m^3^ at 18–22°C and at a relative humidity of 20–50% RH for a duration of 7 days. The measurement error of the Corentium Home (Airthings) detector was 4.95% [21].

### 2.5. Statistical Hypothesis Testing

The normality test for the radon concentration values was conducted for both data sets of provinces and floor levels. The test results of both measurements were found to be normally distributed, and therefore, the arithmetic mean and standard deviation were calculated.

Ten hypotheses were statistically tested to explain the variations:

1. Between the basement floor level (B1) and other floor levels (GF, F1, F2, F3, F4).
2. Between various floor levels (GF, F1, F2, F3, F4).
3. Between the basement floor levels (B1) of Russeifa and the basement floor levels (B1) of other Jordanian provinces.
4. Between various geographic locations of Jordan provinces' basement floor level (B1).
5. Between the floor levels (B1, GF, F1, F2, F3, F4) of Russeifa and those of other Jordanian provinces.
6. Between various geographic locations of Jordan provinces' floor levels (GF, F1, F2, F3, F4).
7. Between a previous study's [6] lower range values and the reported results of this survey for basement level (B1).
8. Between a previous study's [6] lower range values and the reported results of this survey for floor levels (B1, GF, F1, F2, F3, F4).
9. Between a previous study's [6] higher range values and the reported results of this survey for basement level (B1).
10. Between a previous study's [6] higher range values and the reported results of this survey for floor levels (B1, GF, F1, F2, F3, F4).

## 3. Results

### 3.1. Mean Indoor Radon Concentrations in Dwellings

**Figure 1:**
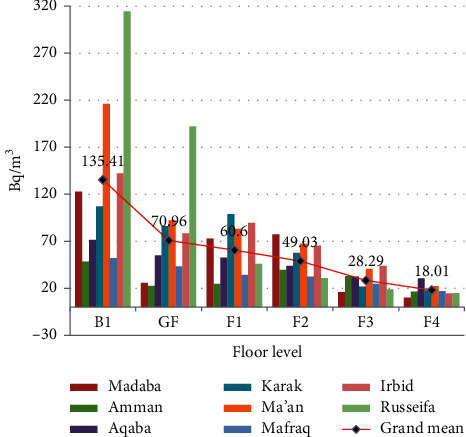
Indoor radon-222 concentration levels of Jordanian cities representing each floor level. The solid line represents the grand mean of the mean values of each floor level.

The results of mean radon concentrations for each floor levels of different Jordanian provinces are presented in Table 1 and Figure 1.

### 3.2. Dose Estimation

**Figure 2:**
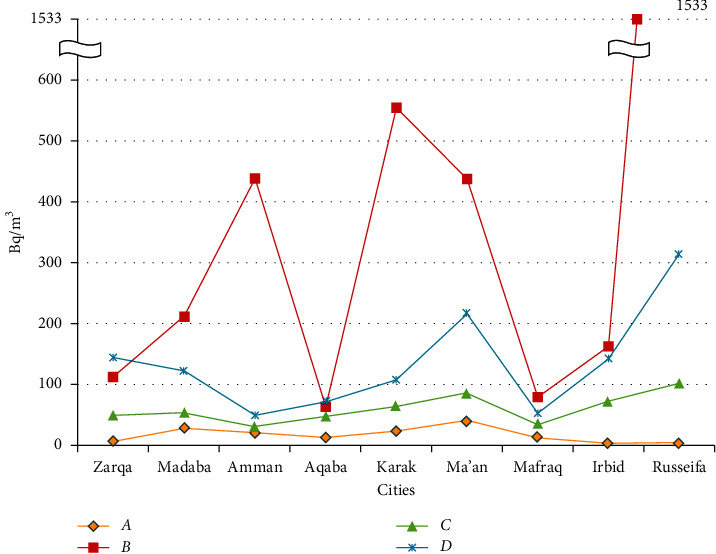
A comparison of the grand mean values of radon concentrations between the floor levels and the basement level of this study to that lower and higher reported values in the literature for various Jordanian cities. *A* is the lower measured values of the reported literature; *B* is the higher measured values of the reported literature; *C* is the grand mean values of floor levels of this study; and *D* is the grand mean values of basement level of this study.

The comparison of dose estimation results of our study and the literature are presented in Table 2 and Figure 2.

## 4. Discussion

The main purposes of this study are to measure the mean values of ^222^Rn gas concentrations for various Jordanian provinces' dwellings and to determine the dose levels among inhabitants. These values are statistically treated and compared with the literature values.

### 4.1. Indoor ^222^Rn Concentrations in Dwellings

The results of this study cover dwellings of the most populated Jordan provinces. This study is intended to shape a clear understanding of the local potential map of alpha energy concentration levels due to ^222^Rn and its progeny based on the distribution of radon gas concentration in various six-floor-level dwellings in each province. The selected six floor levels were the basement (B1), the ground floor (GF), the first floor (F1), the second floor (F2), the third floor (F3), and the fourth floor (F4).

The normality test for the radon concentration values was conducted for both data sets of provinces and floor levels. The test results of both measurements were found to be normally distributed; therefore, the arithmetic mean and one standard deviation have been calculated (Table 1).

Table 1 shows the highest and lowest measured values, i.e., 314.7 Bq/m^3^ at the Russeifa province in a basement (B1) dwelling level and 10.0 Bq/m^3^ at the Madaba province in a fourth (F4) dwelling level, respectively. The pooled mean of the grand mean values for all measurements is 60.4 Bq/m^3^ with the standard deviation equal to 56.2 Bq/m^3^. The ^222^Rn concentration observed in the B1 at each province is found to decrease as follows: Russeifa > Ma'an > Zarqa ≈ Irbid > Madaba > Karak > Aqaba > Mafraq ≈ Amman.

The mean value of indoor radon gas concentrations for the basement level (B1) of Russeifa, which consists of abundant phosphate mines, compared with that of other Jordanian provinces' basement level (B1) is found to be significantly different at *P* < 0.05 [6], whereas mean values of radon gas concentrations in other geographic locations of Jordan provinces' basement level (B1) are not significantly different at *P* < 0.05 (Figure 1).

The mean value of indoor radon concentrations for basement level (B1) compared with other mean values of floor levels (GF, F1, F2, F3, F4) are found to be significantly different at *P* < 0.05. It was also found that there is no significant relation within floor levels (GF, F1, F2, F3, F4) above B1 [9]. This is in agreement with the concept of the main source of the radon gas is seepage from underground rather than from dwelling construction materials.

Furthermore, Figure 1 shows that the mean value of each floor level at any province decreases as the floor level increases.

The mean value of (B1, GF, F1, F2, F3, F4) of Russeifa compared with the mean value of (B1, GF, F1, F2, F3, F4) of other Jordanian provinces has been found to be not significantly different because the predominant source of radon gas has only a slight effect on these mean values.

The mean value of indoor radon concentrations for the basement level (B1) of our study and the lower and higher mean values of radon concentration of previous studies (Table 2) are found to be significantly different at *P* < 0.05 [6, 22]. The mean value of indoor radon concentrations for B1, GF, F1, F2, F3, and F4 of our study and the lower and higher mean values of previous studies are found significantly different at *P* < 0.05 [6, 22].

Figure 2 shows that the mean values of this study lies within the lower and the higher values of the previous measurements for all the provinces. Similarly, the B1 values of this study also lie within the lower and the higher values of the previous measurements for all the provinces.

However, the generally accepted action level established by the World Health Organization (WHO) is 100 Bq/m^3^; with an upper limit that should not exceed 300 Bq/m^3^ [2]. The rounded mean value of B1 concentration levels due to ^222^Rn and its progeny in Zarqa, Madaba, Karak, Ma'an, Irbid and Russeifa are found to be above the accepted action level, whereas the provinces of Amman, Aqaba and Mafraq are found to be below. However, the rounded mean values of floor levels (B1, GF, F1, F2, F3, F4) for Russeifa was also found to be above the upper limit of 300 Bq/m^3^. A recent study by Giraldo-Osorio *et al.* (2020) stated that selected countries across central and South America has indoor limits up to 600 Bq/m^3^, which is well above the upper limit of the action level [23].

### 4.2. Dose Estimation

In order to estimate the received radiation dose by the inhabitants, the annual effective doses *E*(mSv) have been estimated using the occupancy factor of 0.8 (7000 hours per year) and the equilibrium factor of 0.4 for indoor radon in dwellings (ICRP, 1993) [6, 9, 14, 18]. The estimation was only for the measured mean values, of both the basement level (B1) and the floor levels (B1, GF, F1, F2, F3, F4) for each province, as shown in Table 2.

The ^222^Rn effective dose rates in B1 and the mean of floor levels (B1, GF, F1, F2, F3, F4) for each province studied range from 1.2 to 7.9 mSv/year and 0.8 to 2.6 mSv/year, respectively.

In Publication 65 (ICRP, 1993), it is recommended to use the range of about 3–10 mSv/year as a basis for adopting action levels for intervention in dwellings. These values are based on various parameters depending on *F*_*eq*_ and the exposure time. However, Publication 60 (ICRP, 1993) recommended that the effective dose for public exposure should not exceed 1 mSv/year.

The rounded B1 values of exposure to alpha energy concentration levels due to ^222^Rn and its progeny in Zarqa, Madaba, Karak, Ma'an, Irbid, and Russeifa were found to be within the range reported by ICRP (3–10 mSv/year) and for the provinces of Amman, Aqaba, and Mafraq were found to be below the range. However, the rounded mean values of floor levels (B1, GF, F1, F2, F3, F4) for all provinces were found to be below the range of 3–10 mSv/year. In order to minimize the excess exposure to radon gas and its progeny, it is highly recommended to have appropriate ventilation in dwellings for all seasons together with frequent monitoring.

## 5. Conclusion

Radon and its progeny are a health hazard, along with other factors such as living in the basement and without adequate ventilation. Thus, these factors could be lowered and the subsequent hazard could be reduced by appropriate ventilation. All the estimated values of the effective dose in this study are below or within the recommended limit 3–10 mSv/year (ICRP, 1993). However, the recommended effective dose for public exposure by the ICRP, WHO, and IAEA is 1 mSv/year. There are difficulties in estimating the individual effective dose due to uncertainties concerning the stated factors besides medical and other background radiation exposure.

Jordan has a mixed desert/Mediterranean climate, and hence, although it shows marked differences in temperature throughout the year, it is not susceptible to extreme differences in humidity. However, any further investigations could consider any seasonal changes.

Regarding the ^222^Rn concentration in Jordanian dwellings, there are several variations in the acceptable effective limit of 100 Bq/m^3^ and the upper limit of 300 Bq/m^3^.

The decay process of ^222^Rn takes less than 4 days in order to decay to ^210^Pb with a half-life of 22.3 years; having such a toxic element accumulated in the lungs due to continuous breathing of the air contaminated with ^222^Rn gas, this could lead to serious health issues, which require further investigations.

## Figures and Tables

**Table 1:** The mean, grand mean, and pool mean results of radon concentrations in dwellings of Jordanian cities.

	Bq/m^3^									
Floor no.	Zarqa	Madaba	Amman	Aqaba	Karak	Ma'an	Mafraq	Irbid	Russeifa	Grand mean^a^
B1	143.9	122.8	48.3	71.3	107.0	**216.3**	52.2	142.2	314.7	**135.4** **±** 85.4
GF	42.9	25.7	22.7	54.9	86.2	92.2	43.4	78.3	192.4	**71.0** **±** 52.0
1	43.3	72.9	24.8	52.5	98.8	83.4	34.2	89.7	45.9	**60.6** **±** 26.3
2	26.6	77.4	39.6	43.9	57.7	67.4	32.5	65.3	30.7	**49.0** **±** 18.4
3	23.7	15.9	32.9	32.3	21.8	40.7	24.7	43.8	18.9	**28.3** **±** 9.7
4	18.9	10.0	16.4	30.6	17.8	22.4	16.9	14.4	14.8	**18.0** **±** 5.8
Grand mean^b^	**49.9** **±** 47.2	**54.1** **±** 44.3	**30.8** **±** 11.8	**47.6** **±** 15.4	**64.9** **±** 38.8	**87.1** **±** 68.5	**34.0** **±** 12.7	**72.3** **±** 43.5	**102.9** **±** 123.4	**60.4** **±** 56.2^c^

^a^Grand arithmetic mean and its one sigma standard deviation for each floor level for all cities. ^b^Grand arithmetic mean and its one sigma standard deviation for each city for all floor levels. ^c^Pool arithmetic mean and its one sigma standard deviation for all floor levels in all cities.

**Table 2:** Summary of current measurement results of radon concentration and annual effective dose of indoor radon in dwelling buildings for the mean values of floor levels (B1, GF, F1, F2, F3, F4) and for the basement level (B1) compared with the literature-reported values of lower range and higher ranges for various Jordanian cities.

City	Literature-reported lower values Bq/m^3^	Literature-reported higher values Bq/m^3^	Current measurement (B1, GF, F1, F2, F3, F4) Bq/m^3^	Current measurement (B1) Bq/m^3^	Current measurement of effective dose (B1, GF, F1, F2, F3, F4) mSv/year	Current measurement of effective dose (B1) mSv/year
Zarqa	6.9	113.1	49.9	143.9	1.3	3.6
Madaba	28	212	54.1	122.8	1.4	3.1
Amman	20	440	30.8	48.3	0.8	1.2
Aqaba	12	64	47.6	71.3	1.2	1.8
Karak	24	556	64.9	107.0	1.6	2.7
Ma'an	40	440	87.1	216.3	2.2	5.5
Mafraq	12	80	34.0	52.2	0.9	1.3
Irbid	3.1	163.9	72.3	142.2	1.8	3.6
Russeifa	4	1532.9	102.9	314.7	2.6	7.9
Grand mean^a^	**16.7** **±** **12.4**	**400.2** **±** **460.9**	**60.4** **±** **56.2**	**135.4** **±** **85.4**	**1.5** **±** **0.6**	**3.4** **±** **2.2**

^a^Grand arithmetic mean and its one sigma standard deviation for each Jordanian province.

## Data Availability

The data used to support the study are available from the corresponding author upon request.
